# TFAM signaling molecule alleviates mitochondrial damage of cerebral ischemia-reperfusion

**DOI:** 10.1038/s41420-025-02930-x

**Published:** 2026-01-08

**Authors:** Wenjun Wang, Yibiao Shi, Sitian Qiu, Ying Song, Xi Chen, Xiaomin Zhang, Beibei Wang, Qisong Li, Qiwen Shi

**Affiliations:** 1 Department of Pharmacology, Zhejiang University of Technology, Hangzhou,, 310014 Zhejiang China; 2 Hangzhou King’s Bio-pharmaceutical Technology Co., Ltd., Hangzhou, Zhejiang China; 3 The First People’s Hospital of Linping District, Hangzhou, Zhejiang China; 4 Chongqing Industry and Information Vocational College, Chongqing, China

**Keywords:** Molecular neuroscience, Energy metabolism

## Abstract

In the present study, we aimed to investigate the antioxidant and therapeutic protective effects of carvacryl acetate (CAA) on Mitochondrial damage of cerebral ischemia-reperfusion through mitochondrial transcription factor A (TFAM) signaling molecules.SD rats were used to establish the middle cerebral artery occlusion (MCAO) model in vivo, and PC12 cells were stimulated with H_2_O_2_ in vitro. Longa neurological score and triphenyltetrazolium chloride (TTC) staining was used to observe the ischemic infarction. Transmission electron microscope (TEM) was used to observe the mitochondria. Reactive Oxygen Species/ Superoxide Dismuptase/Malondialdehyde/Adenosine Triphosphate (ROS/SOD/MDA/ATP) detection kit was used to detect. RT-qPCR was used to detect the mRNA level of target gene and mitochondrial DNA (mtDNA) copy number changes. Immunofluorescence and Western blot were used to detect the expression of protein. After oxidative stress in the MCAO model of SD rats, the neurological score increased, the volume of ischemic area of cerebral infarction increased, the morphology of nerve cells in brain tissue and PC12 cells was disordered, the mitochondria appeared vacuolated, the contents of ROS and MDA increased, and the activity of SOD decreased. Oxidative stress causes mitochondrial dysfunction, resulting in the reduction of mtDNA copy number and the decreased expression of TFAM in brain tissue nerve cells and PC12 cells, which in turn affects mitochondrial transcription biogenesis and decreases the expression of POLRMT and TFB_2_M molecules. CAA promotes intracellular TFAM expression and activates its antioxidant pathway, thereby protecting mtDNA and alleviating oxidative stress and mitochondrial damage caused by MCAO in vivo and H_2_O_2_ stimulation in vitro. Lentivirus downregulates the expression of TFAM, and under its action, the antioxidant and mitochondrial protection effects of CAA are weakened. When TFAM was disrupted, the protective effect of CAA on mitochondria was inhibited. Compared to edaravone, a positive control, CAA exhibited similar therapeutic effects. These findings suggest that CAA alleviates CIRI through TFAM signaling pathways, offering potential therapeutic implications for ischemic stroke treatment.

## Introduction

Stroke is divided into two types: ischemic stroke and hemorrhagic stroke [1]. It is a disease caused by various reasons, resulting in focal or global brain tissue damage. Therefore, restoring blood flow as soon as possible is the goal of treatment [2]. However, with the rapid increase in blood flow, a large amount of reactive oxygen species (ROS) is produced, causing damage to mitochondrial function, which is known as cerebral ischemia-reperfusion injury (CIRI) [3, 4]. Among them, oxidative stress is one of the key mechanisms closely related to the pathophysiology of CIRI [5, 6].

Mitochondria, a two-layer membrane-coated organelle present in most cells, are the main site of aerobic respiration in cells, and their function plays a crucial role in maintaining the homeostasis of the body [7, 8]. TFAM (mitochondrial transcription factor A), a nuclear gene encoding mitochondrial transcription factor, is a DNA-binding protein and an important factor in maintaining the normal function of mtDNA [9]. It can not only stabilize mtDNA but also initiate mtDNA replication, which plays a crucial role in mtDNA metabolism [10]. The relationship between TFAM and mitochondria is reciprocal. TFAM can protect mitochondrial DNA from ROS attack, while mitochondrial DNA protects TFAM from degradation by Lon protease [11, 12].

Mitochondrial biogenesis refers to a regenerative program that maintains mitochondrial numbers, replacing old and damaged mitochondria with new and healthy ones [13]. Among them, the related factors of mitochondrial biogenesis include TFAM, POLRMT, TFB_2_M, etc. Mitochondrial transcription combines with mtDNA distortion and recruits POLRMT to form a ternary complex, and then recruits TFB_2_M to form a quaternary complex, thus leading to mitochondrial transcription biogenesis [14].

Carvacryl acetate (CAA) and ED are obtained by acetylation of carvacrol, and in the medical and biological fields, several studies have shown that CAA shows higher antioxidant activity compared with other common volatile components of essential oils [15]. Studies have shown that CAA has a wide range of antibacterial, anti-inflammatory, analgesic, and antioxidant activities [16], and also shows strong anti-epileptic, anti-anxiety, and brain function protection in the central nervous system [17, 18]. CAA has a protective and antioxidant effect on the body in terms of oxidative stress, which is related to mitochondrial function, but there are few studies on the conduction of TFAM signaling molecules.

In the present study, for the first time, we used the model of CIRI induced by middle cerebral artery occlusion (MCAO) in vivo and the model of oxidative stress induced by H_2_O_2_ in SD rats in vitro, and combined with shTFAM lentivirus to explore the antioxidant effect of CAA/ED and the mechanism related to TFAM signaling molecules.

## Results

### CAA/ED can alleviate CIRI in SD rats

#### CAA/ED can reduce neurological scores and cerebral infarction volume

**Fig. 1:**
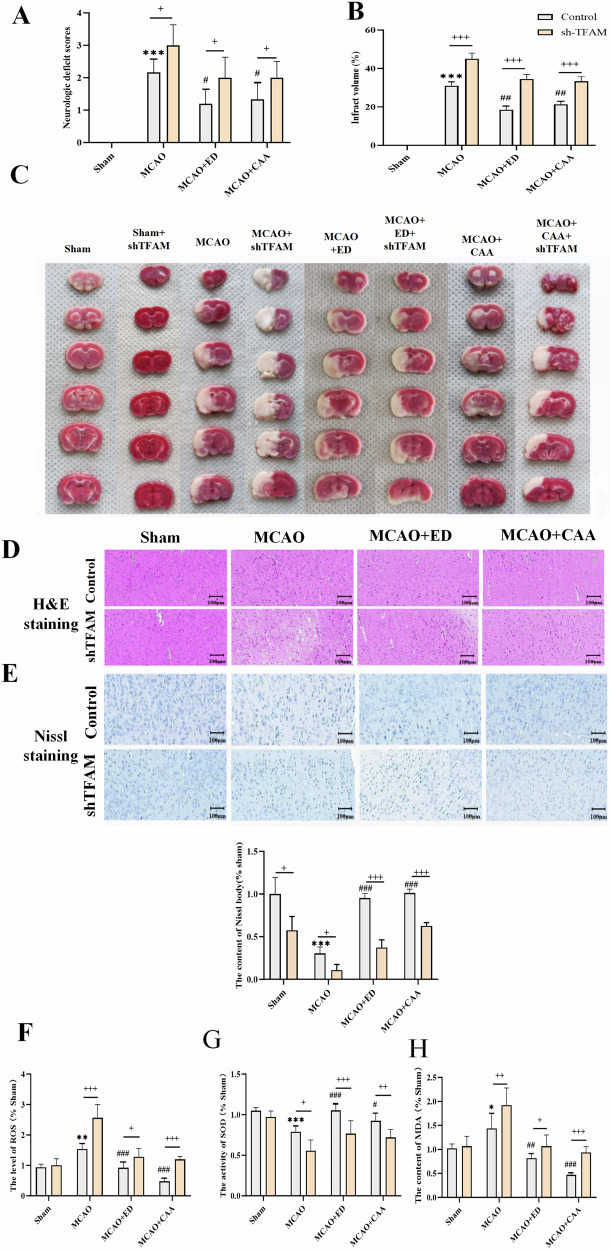
CAA/ED can alleviate CIRI in SD rats. Sham group: control group; sh-TFAM: TFAM down-regulated group; MCAO: cerebral ischemia reperfusion model group; ED: Edaravone administration group; CAA: Carvacrol acetate administration group. **A** Neurological scores of SD rats (*n* = 8). **B** Analysis of TTC cerebral infarction in SD rats (*n* = 3). **C** TTC was used to observe cerebral infarction in SD rats. **D** HE staining in SD rats. **E** Nissl staining in SD rats. **F** ROS levels in brain tissue (*n* = 6). **G** SOD activity in brain tissue (*n* = 6). **H** MDA content in brain tissue (*n* = 6). ^*^P < 0.05, ^**^P < 0.01, ^***^P < 0.001 vs. Sham; ^#^*P* < 0.05, ^##^*P* < 0.01, ^###^P < 0.001 vs. MCAO; ^+^*P* < 0.05, ^++^*P* < 0.01, ^+++^*P* < 0.001 vs. sh-TFAM.

The neurological scores of the in vivo experiment (Fig. 1A) showed that the neurological scores of the MCAO group increased compared with the Sham group, and the neurological scores of the drug group decreased, while the symptoms of the shTFAM + MCAO group worsened. The therapeutic effect of the shTFAM + drug group was lower than that of the normal drug group, and the normal experimental group and the shTFAM experimental group had statistically significant differences (*P* < 0.05). TTC staining was used to observe the infarct area of brain tissue in rats (Fig. 1B, C). Compared with the Sham group, the cerebral infarction area in the MCAO group was significantly increased, and the infarct area was reduced after CAA/ED treatment, but the cerebral infarction area in the shTFAM group was significantly increased, and the therapeutic effect of drug was also inhibited accordingly. There was a significant difference between the normal experimental group and the shTFAM experimental group (*P* <0.001).

#### CAA/ED can improve the pathological changes of brain tissue induced by CIRI

The results of hematoxylin-eosin staining (H&E) and Nissl staining of brain tissue sections showed (Fig. 1D) that the morphology of nerve cells in the Sham group was clearly visible, and the cells were densely arranged, while the number of nerve cells in the model group was reduced, the cells were arranged disorderly, and the cell body was atrophied. After drug treatment, the degree of injury was alleviated. However, the degree of cell injury was increased in the shTFAM group, and the degree of remission was weakened by drug treatment.

#### CAA can alleviate oxidative stress injury induced by CIRI

The results of oxidative stress indexes in brain tissue showed that the ROS level and Malondialdehyde (MDA) content in MCAO group were higher than those in Sham group, and the superoxide dismuptase (SOD) activity was decreased. After CAA treatment, all three indexes were improved. The degree of oxidative stress was aggravated in shTFAM + MCAO group, and the relief effect of shTFAM + drug group was lower than that of normal drug group (Fig. 1E–G).

#### CAA/ED could reduce mitochondrial morphological damage induced by CIRI

**Fig. 2:**
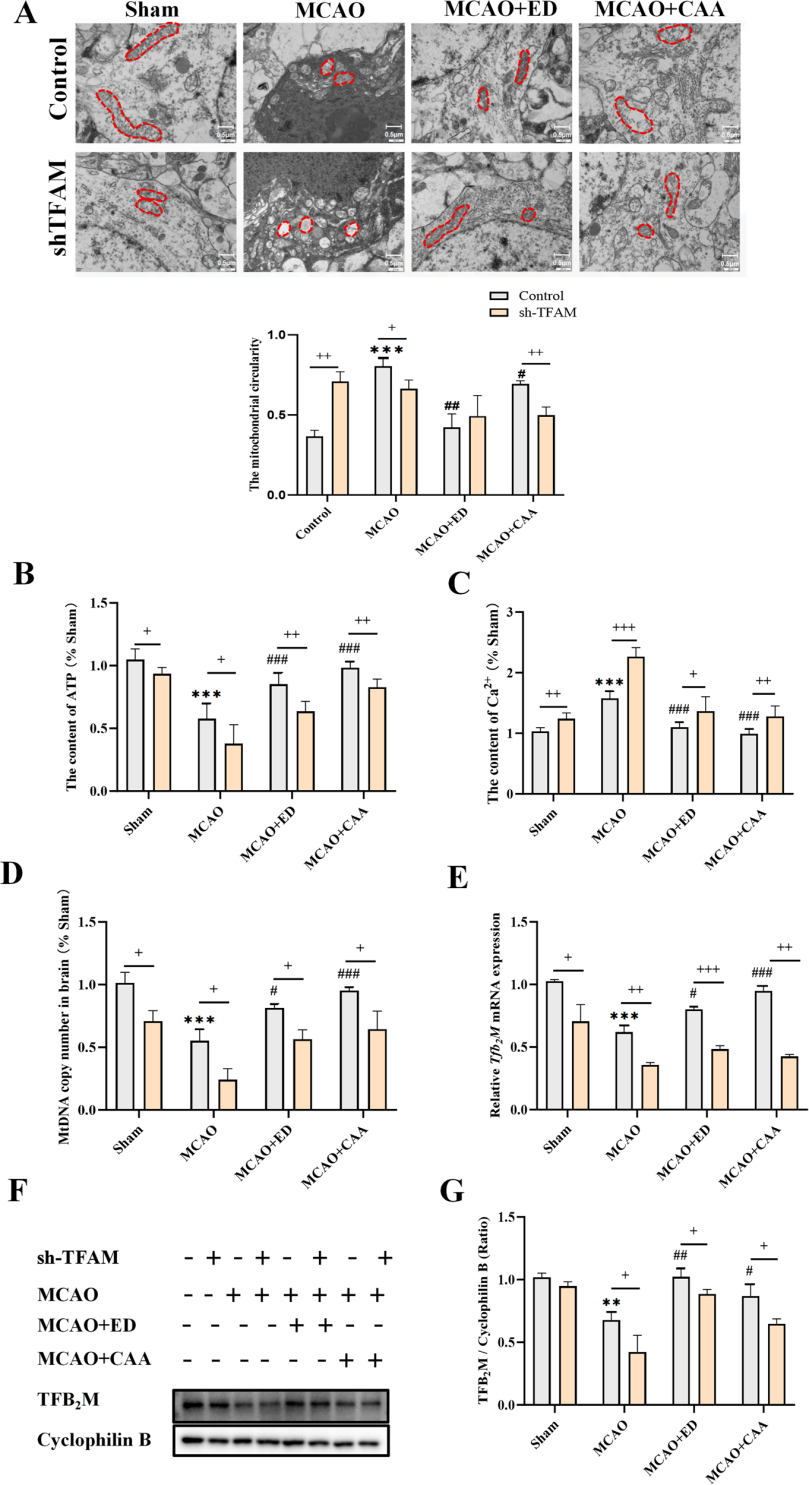
CAA/ED can improve mitochondrial dysfunction to alleviate CIRI. Sham group: control group; sh-TFAM: TFAM down-regulated group; MCAO: cerebral ischemia reperfusion model group; ED: Edaravone administration group; CAA: Carvacrol acetate administration group. **A** The morphology of mitochondria in SD rats was observed by transmission electron microscopy and statistics. **B** ATP content in brain tissue (*n* = 6). **C** Brain Ca^2+^ content (*n* = 6). **D** mtDNA copy number in brain tissue (*n* = 3). **E** Tfb_2_M mRNA levels in brain tissues (*n* = 3). **F** TFB_2_M protein content in brain tissue (*n* = 3). **G** Analysis of TFB_2_M protein ratio in brain tissue (*n* = 3). ^**^P < 0.01, ^***^P < 0.001 vs. Sham; ^#^*P* < 0.05, ^##^*P* < 0.01, ^###^*P* < 0.001 vs. MCAO; ^+^*P* < 0.05, ^++^*P* < 0.01, ^+++^*P* < 0.001 vs. sh-TFAM.

By observing the morphology of mitochondria in tissue cells under TEM (Fig. 2A), compared with the Sham group, the mitochondria in the model group were damaged, which was manifested as a decreased number of mitochondria, swelling of mitochondria, and an increased degree of vacuolization. After drug treatment, the degree of mitochondrial damage was alleviated, some mitochondria were recovered, and the number of damaged mitochondria was reduced. However, in the shTFAM + MCAO group, the degree of mitochondrial damage was aggravated, the mitochondrial cristae disappeared, and the therapeutic effects of shTFAM + ED and CAA drugs were also partially inhibited.

#### CAA/ED could improve the changes of ATP content and Ca^2+^ content induced by CIRI

The results of ATP content and Ca^2+^ content detection showed (Fig. 2B, C) that compared with the Sham group, the ATP content decreased, and Ca^2+^ content increased, mitochondrial function damage increased, and membrane permeability increased in the MCAO model group. After ED and CAA drug treatment, ATP content increased significantly, and Ca^2+^ content decreased significantly. However, in the shTFAM group, mitochondrial function and membrane permeability were aggravated.

#### CAA/ED can regulate mtDNA copy number and TFB_2_M expression

The detection of mtDNA copy number in brain tissue showed that the mtDNA copy number in the model group would decrease due to mitochondrial dysfunction, and this phenomenon was relieved and restored after the treatment with ED and CAA drugs (Fig. 2D). However, when TFAM was interfered, mtDNA copy number decreased significantly, and the therapeutic effect of drugs was also partially inhibited. The results of RT-qPCR and Western blot analysis of TFB_2_M signaling molecules (Fig. 2E–G) showed that *Tfb*_*2*_*M* mRNA level and protein expression were decreased, and their expression was increased by ED and CAA administration. It may be suggested that CAA may alleviate oxidative stress injury by regulating the expression of mitochondrial transcription biogenesis genes. After shTFAM lentivirus treatment, the expression of TFB_2_M in the model group was significantly decreased, and the increase of TFB_2_M in the drug group was also partially inhibited.

#### CAA/ED can regulate TFAM and POLRMT

**Fig. 3:**
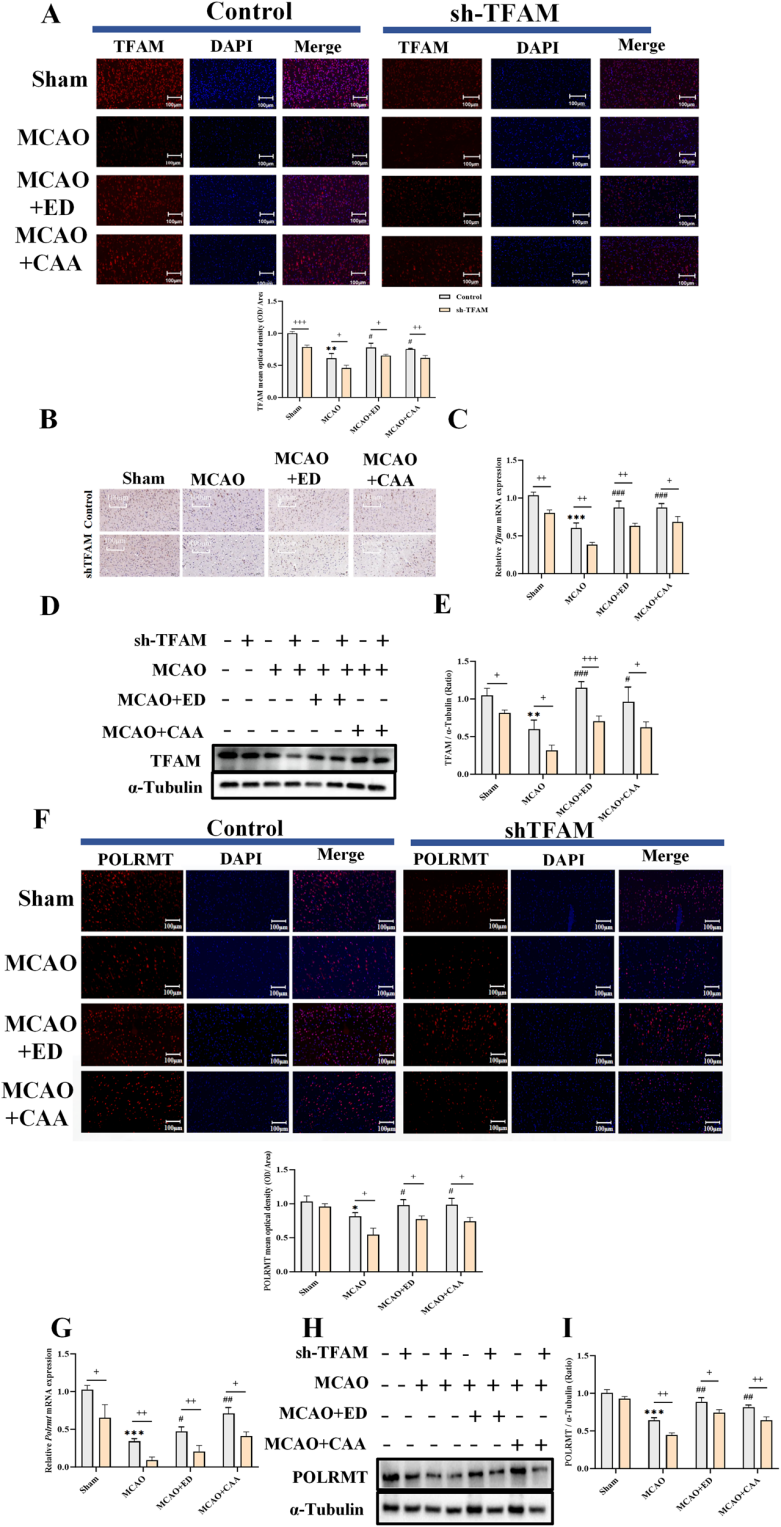
CAA regulates mitochondrial transcription of biogenesis genes to alleviate CIRI. Sham group: control group; sh-TFAM: TFAM down-regulated group; MCAO: cerebral ischemia reperfusion model group; ED: edaravone administration group; CAA: carvacrol acetate administration group. **A** Immunofluorescence and ratio analysis of TFAM in cerebral cortex of brain tissue (*n* = 3). **B** Immunohistochemistry of TFAM in cerebral cortex of brain tissue. **C** Tfam mRNA levels in brain tissues (*n* = 3). **D** TFAM protein content in brain tissue (*n* = 3). **E** Analysis of TFAM protein ratio in brain tissue (*n* = 3). **F** Immunofluorescence and ratio analysis of POLRMT in cerebral cortex (*n* = 3). **G** Polrmt mRNA levels in brain tissues (*n* = 3). **H** POLRMT protein content in brain tissue (*n* = 3). **I** Analysis of POLRMT protein ratio in brain tissue (*n* = 3).^*^P < 0.05, ^**^P < 0.01, ^***^P < 0.001 vs. Sham; ^#^*P* < 0.05, ^##^*P* < 0.01, ^###^*P* < 0.001 vs. MCAO; ^+^*P* < 0.05, ^++^*P* < 0.01, ^+++^*P* < 0.001 vs. sh-TFAM.

We detected TFAM and POLRMT in the brain tissue of SD rats by immunofluorescence technology, and detected TFAM expression in the brain tissue by immunohistochemistry. The results of TFAM and POLRMT expression analysis by RT-qPCR and Western blot showed that (Fig. 3A–I), the expression of TFAM and POLRMT decreased in the MCAO model group, and increased after drug treatment. However, after shTFAM lentivirus treatment, the expression of TFAM and POLRMT decreased significantly (*P* < 0.05, *P* < 0.01).

### CAA/ED can alleviate H_2_O_2_-induced oxidative stress in PC12 cells

#### Selection of optimal drug concentration

**Fig. 4:**
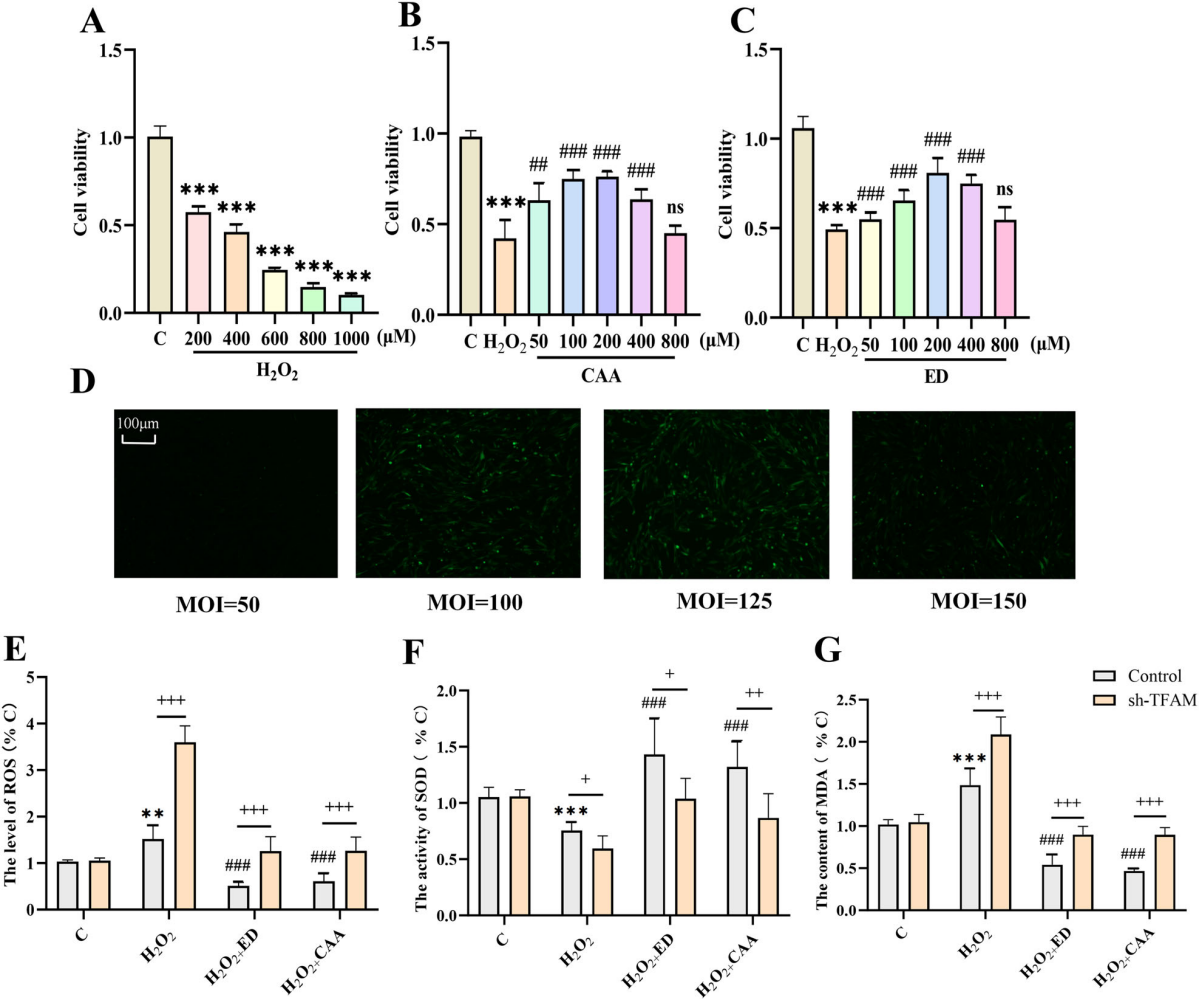
CAA/ED can alleviate oxidative stress injury induced by H_2_O_2_. Control: control group; sh-TFAM: TFAM down-regulated group; H_2_O_2_: oxidative stress stimulation group; ED: Edaravone administration group; CAA: Carvacrol acetate administration group. **A** Effect of H_2_O_2_ concentration on PC12 cell survival (*n* = 6). **B** Effect of CAA concentration on PC12 cell survival (*n* = 6). **C** Effect of ED concentration on PC12 cell survival (*n* = 6). **D** Fluorescence pattern of PC12 cells transfected with shTFAM at different MOI concentrations. **E** ROS levels in PC12 cells (*n* = 6). **F** SOD activity in PC12 cells (n = 6). **G** MDA content in PC12 cells (*n* = 6). ^**^P < 0.01, ^***^P < 0.001 vs. C; ^###^*P* < 0.001 vs. H_2_O_2_; ^+^*P* < 0.05, ^++^*P* < 0.01, ^+++^*P* < 0.001 vs. sh-TFAM.

According to the MTT results, the working concentration of H_2_O_2_ was selected as 400 μM, the working concentration of CAA was 100 μM, and the working concentration of ED was 200 μM (Fig. 4A–C). According to the fluorescence results of shTFAM-transfected PC12 cells, we finally selected MOI = 125 as our experimental condition for subsequent experiments (Fig. 4D).

#### CAA/ED can alleviate oxidative stress injury induced by H_2_O_2_

The results of oxidative stress indexes of PC12 cells showed that the ROS level and MDA content in the H_2_O_2_ group were higher than those in the Control group, and the SOD activity was lower than that in the control group (Fig. 4E–G). After CAA/ED treatment, the three indexes were improved, and the results were consistent with the in vivo experiments. After downregulation of TFAM by lentivirus, oxidative stress was aggravated in the shTFAM + model group.

#### CAA/ED could reduce mitochondrial morphological damage induced by H_2_O_2_

The results of TEM showed that compared with the control group, the mitochondria in the model group were damaged, while after drug treatment, the degree of mitochondrial damage was alleviated, some mitochondria were recovered, and the number of damaged mitochondria was reduced. However, in the shTFAM group, the degree of mitochondrial damage was aggravated, the disappearance of mitochondrial cristae and the degree of vacuolization were aggravated, and the therapeutic effects of ED and CAA drugs were also partially inhibited.

#### CAA/ED can improve the changes of ATP content and Ca^2+^ content induced by H_2_O_2_

The results of the detection of ATP content and Ca^2+^ content in PC12 cells showed that, compared with the Control group, the ATP content in the H_2_O_2_ model group decreased, and the Ca^2+^ content increased, and both were improved after CAA treatment. However, when TFAM was downregulated, the ATP content in the H_2_O_2_ model group decreased significantly, and the Ca^2+^ content increased significantly, and the improvement effect of the drug was weakened.

#### CAA/ED could alleviate H_2_O_2_-induced mtDNA copy number and TFB_2_M expression

**Fig. 5:**
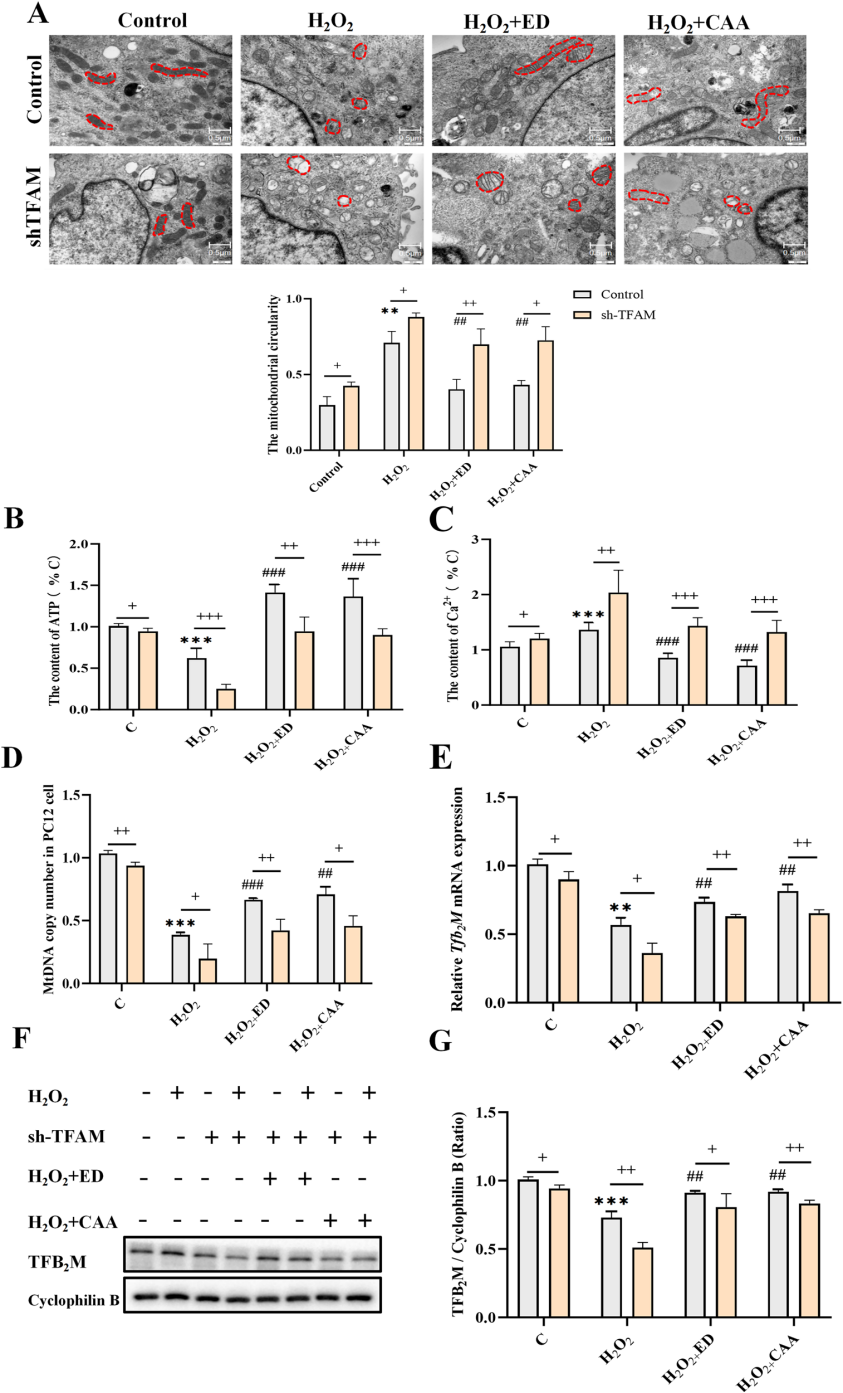
CAA/ED can ameliorate mitochondrial dysfunction to alleviate H_2_O_2_ -induced oxidative stress injury. Control: control group; sh-TFAM: TFAM down-regulated group; H_2_O_2_: oxidative stress stimulation group; ED: Edaravone administration group; CAA: Carvacrol acetate administration group. **A** Mitochondrial morphology of PC12 cells was observed by TEM transmission electron microscope. **B** ATP content of PC12 cells (*n* = 6). **C** Ca^2+^ content of PC12 cells (*n* = 6). **D** MtDNA copy number of PC12 cells (*n* = 3). **E** Tfb_2_M mRNA levels in PC12 cells (*n* = 3). **F** TFB_2_M protein content in PC12 cells (*n* = 3). **G** Analysis of TFB_2_M protein ratio in PC12 cells (*n* = 3). ^**^P < 0.01, ^***^P < 0.001 vs. C; ^##^*P* < 0.01, ^###^*P* < 0.001 vs. H_2_O_2_; ^+^*P* < 0.05, ^++^*P* < 0.01, ^+++^*P* < 0.001 vs. sh-TFAM.

The detection of mtDNA copy number in brain tissue showed that the mtDNA copy number of the model group decreased, while the mtDNA copy number of the CAA drug group increased. When TFAM was interfered, mtDNA copy number decreased significantly, suggesting a close relationship between the therapeutic mechanism of CAA and mtDNA copy number. Subsequently, TFB_2_M signaling molecules were detected by RT-qPCR and Western blot. As shown in Fig. 5E–G, the mRNA level and protein expression of TFB_2_M in the PC12 cell model group were significantly lower than those in the Control group. The mRNA and protein expression of TFB_2_M in the ED and CAA drug groups were higher than those in the H_2_O_2_ group, which was consistent with the in vivo results. After shTFAM lentivirus treatment, the expression of TFB_2_M in the model group was significantly lower than that in the normal group.

#### CAA/ED could regulate the expression of TFAM and POLRMT induced by H_2_O_2_

**Fig. 6:**
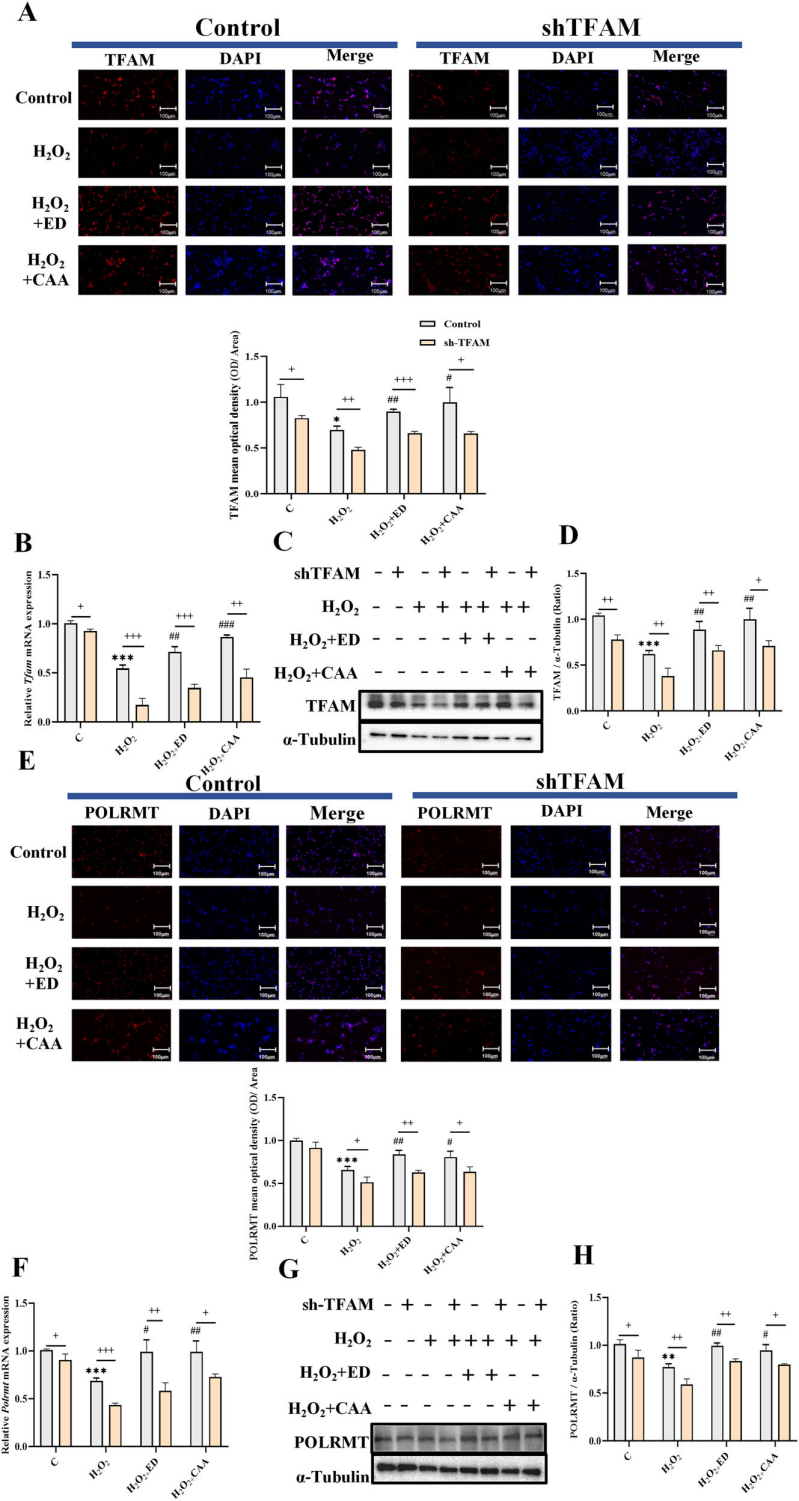
CAA/ED can modify mitochondrial pathway biogenesis genes to alleviate oxidative stress injury induced by H_2_O_2_. Control: control group; sh-TFAM: TFAM down-regulated group; H_2_O_2_: oxidative stress stimulation group; ED: Edaravone administration group; CAA: Carvacrol acetate administration group. **A** TFAM immunofluorescence and ratio analysis in PC12 cells (*n* = 3). **B** Tfam mRNA levels in PC12 cells (*n* = 3). **C** TFAM protein content in PC12 cells (*n* = 3). **D** Analysis of TFAM protein ratio in PC12 cells (*n* = 3). **E** POLRMT immunofluorescence and ratio analysis in PC12 cells (n = 3). **F** Polrmt mRNA levels in PC12 cells (*n* = 3). **G** POLRMT protein content in PC12 cells (*n* = 3). **H** Analysis of POLRMT protein ratio in PC12 cells (*n* = 3). ^*^P < 0.05, ^**^P < 0.01, ^***^P < 0.001 vs. C; ^#^*P* < 0.05, ^##^*P* < 0.01, ^###^*P* < 0.001 vs. H_2_O_2_; ^+^*P* < 0.05, ^++^*P* < 0.01, ^+++^*P* < 0.001 vs. sh-TFAM.

TFAM and POLRMT in PC12 cells were detected by immunofluorescence as well as RT-qPCR and Western blot, and the results shown in Fig. 6A–G showed that the expression of TFAM and POLRMT was reduced in the H_2_O_2_ group compared with the control group. Compared with the model group, both expressions were increased. The expression of TFAM and POLRMT in shTFAM lentivirus-transfected group was lower than that in the normal group.

## Experimental methods and materials

### Experimental materials

ED was purchased from Jiangsu Simcere Pharmaceutical Co., LTD. CAA was purchased from EXTRASYNTHESE (France) with a purity above 95%; Fetal bovine serum (FBS) was purchased from Hangzhou Sijiqing Company; Dulbecco's Modified Eagle Medium (DMEM) high glucose medium was purchased from Wuhan Punosai Technology Co., LTD. Rabbit polyclonal TFAM antibody (ab307302) and sheep polyclonal TFB_2_M antibody (ab118321) were purchased from Abcam, USA. Rabbit polyclonal POLRMT (PA5-116630) antibody was purchased from Thermo Fisher Scientific; MCAO wire bolt was purchased from Beijing Xinong Technology Co., LTD. TFAM lentivirus was purchased from Shanghai Jima Pharmaceutical Technology Co., LTD. MDA, SOD and ROS were purchased from Nanjing Jiancheng BioEngineering Institute.

### The model of SD rats

All male SD rats were housed in separate cages in the rat housing room of the Animal Experiment Center of Zhejiang University of Technology. The feeding environment was as follows: quiet, room temperature 21–25 °C, 50% humidity, and 12 h light-dark cycle. SD rats were fed and watered AD libitum.

All surgical procedures related to animals in the experiment were carried out after the approval of the Experimental Animal Ethics Committee of Zhejiang University of Technology. All experimental procedures were conducted in accordance with the Guide for the Care and Use of Laboratory Animals in the Zhejiang University of Technology, Hangzhou, China, and conformed to the National Institutes of Health Guide for Care and Use of Laboratory Animals (Publication No. 85-23, revised 1996). Then, SD rats were anesthetized by intraperitoneal injection of 20% urethane. The scalp was removed, and the head was fixed in a stereotaxic apparatus using ear bars and a holder. The skull was exposed by incising the scalp to reveal the bregma and lambda sutures. With the bregma set as the injection reference point, the coordinates for injection were determined as *X* = +1.5 mm, *Y* = +1.8 mm, and *Z* = −3.5 mm. A cranial drill was used to create a burr hole at the marked injection site. Lentiviral stock solution (2 μL) was injected at a constant rate of 0.2 μL/min. The needle remained in place for 5 min post injection to ensure adequate viral diffusion before slow withdrawal. The wound was sutured and disinfected with iodine. All procedures were performed in a BSL-2 laboratory in strict compliance with biosafety guidelines. MCAO animal experiments were performed. All surgical procedures related to animals in the experiment were carried out after the approval of the Experimental Animal Ethics Committee of Zhejiang University of Technology.

### PC12 cells culture and treatment

PC12 cells were obtained from Institute of Biology, Chinese Academy of Sciences. The cells were cultured in DMEM high glucose medium (containing 10% FBS and 1% anti-penicillin-streptomycin and gentamicin) at 37 °C in 5% CO_2_ incubator. PC12 cells were treated with different concentrations of H_2_O_2_ (200, 400, 600, 800, 1000 μM), ED (50, 100, 200, 400, 800 μM), and CAA (50, 100, 200, 400, 800 μM) for 24 h to explore the drug concentration.

shTFAM cell transfection: (1) Suitable PC12 cells were seeded in 96-well plates. (2) After overnight culture, the original medium was replaced by half the volume of virus working solution containing different MOI (MOI = 50, 100, 125, 150). (3) After 4 h, half volume of dye assistant solution containing 6 μg/ml polybrene was added and incubated at 37 °C. (4) PC12 cells with TFAM knockdown were selected by changing to normal culture medium at 24 h, and culture medium containing 2 μg/ml puromycin was added at 3 days. (5) The transfection efficiency of lentivirus was observed by fluorescence microscope. (6) The optimal MOI of transfection was used for subsequent experiments.

### Experimental grouping

The experiment was divided into two parts, in vivo and in vitro, with 8 groups in each part. In vivo experiment: Sham operation group, Sham + shTFAM group, MCAO group, MCAO + shTFAM group, ED positive drug group, ED + shTFAM group, CAA treatment group, CAA + shTFAM group; In vitro experiment: control group, control + shTFAM group, H_2_O_2_ group, H_2_O_2_ + shTFAM group, ED positive drug group, ED + shTFAM group, CAA treatment group, CAA + shTFAM group.

### MCAO model

The left common carotid artery was exposed after the rats were anesthetized. The vagus nerve and parasympathetic nerve were gently separated, and the common carotid artery and external carotid artery were ligated. The common carotid artery was opened with a V-shaped opening, the thread was inserted into the internal carotid artery, and the thread was fixed. CIRI was achieved by removing the thread plug 90 min later. Normal saline and corresponding drugs were administered at 0 h and 12 h after thrombectomy.

### Neurological score

Behavioral analyzes were performed by a double-blind method. 24 h after cerebral ischemia-reperfusion, the neurological function was evaluated by Longa neurological scoring system.

### TTC

The brain tissue of SD rats was removed and rinsed gently in normal saline, then placed at −20 °C, and then quickly sectioned along the coronal plane of the brain with a sharp blade, with a thickness of about 2 mm. A total of 6 pieces were cut, and an appropriate amount of TTC staining solution was added and placed in a constant temperature oven at 37 °C, and stained in the dark for 30 min to make it in uniform contact with the liquid. Image Pro plus software was used to analyze and calculate the infarct volume of brain tissue. The percentage of infarct volume = infarct volume/infarct side half brain volume × 100%.

### H&E staining

Following cardiac perfusion with 0.9% saline, brain tissues were fixed in 4% paraformaldehyde at 4 °C overnight. Sections were mounted with neutral gum. Results were visualized using scanning software.

### Nissl staining

Paraffin embedding and sectioning steps were performed as H&E staining. Sections were subjected to 95% ethanol for 5 s, absolute ethanol for 1 min, and xylene three times for 2 min each. Scanning software was used to observe the results. Within the target area of each photo, the “Cell Counter” plugin of ImageJ software is used to calculate the number of Nissl positive cells.

### TEM was used to observe mitochondrial morphology

Brain tissues and PC12 cells were fixed in 2.5% glutaraldehyde (PBS) at 4 °C overnight, rinsed with 0.1 M phosphate buffer (3 × 15 min), and post-fixed in 0.1 M osmic acid for 2 h. Mitochondrial morphology was analyzed by TEM. We conducted quantitative analysis of mitochondrial morphology in TEM images. Tissue samples and PC12 cells from three randomly selected animals in each group were processed, with at least three clear TEM images captured per sample. Using ImageJ software, the contours of each clearly distinguishable mitochondrion were manually outlined, excluding those overlapping with surrounding structures or with incomplete cross-sections. The perimeter and area of each mitochondrion were calculated, and the Circularity formula = 4π*Area/(perimeter)² was applied. The closer the result is to 1, the more circular the mitochondria are, and the lower their activity.

### Immunofluorescence staining

Cells grown on cover slides were fixed with 4% paraformaldehyde solution and then blocked for 1 h at room temperature. After blocking, the primary antibody was dropped onto the sections and incubated overnight at 4 °C. Secondary antibodies (Beyotime) were added and incubated for 2 h at 37 °C. Sections were observed under a fluorescence microscope and photographed.

### Detection of oxidative stress indicators

ROS content: DCFH-DA probe was used to load tissue homogenate and cells, with ROS detected by fluorescence microplate reader (Ex: 488 nm, Em: 525 nm). Results were normalized to protein concentration.

SOD activity: Samples were lysed on ice for 15 min, centrifuged at 14,000 × *g* (4 °C, 10 min), and supernatants collected. Protein concentration was measured by bicinchoninic acid (BCA) assay, and SOD activity was determined using a detection kit, normalized to protein concentration.

MDA content: Samples were processed as for SOD, and MDA levels were measured using a detection kit, normalized to protein concentration.

### Mitochondrial index detection

ATP content determination: ATP lysate was added to the homogenate tissue and PC12 cells, and after lysis, the supernatant was removed by centrifugation at 12000 × *g* for 5 min at 4 °C and used for subsequent determination. ATP content in brain tissue and PC12 cells was determined according to the instructions of the Enhanced ATP Assay Kit (Beyotime, Shanghai, China) and normalized by protein concentration.

Ca^2+^ content detection: After the homogenate tissue and PC12 cells were collected, an appropriate amount of detection lysate was added, and the supernatant was collected by centrifugation at 14,000 × *g* for 5 min at 4 °C. Calcium content in tissue homogenates and PC12 cells was determined according to the instructions of the Calcium content chromogenic detection kit (Shanghai Biyuntian Biotechnology Co., LTD) and normalized by protein concentration.

### RT-qPCR and mtDNA copy number

Total RNA from tissues and PC12 cells was extracted using Trizol (Invitrogen, USA, 12183-555) and reverse transcribed using a kit (Invitrogen, USA, 11752-050). Primer sequences (Sangon, Shanghai, China) are shown in Table 1.

**Table 1:** Primers for RT-qPCR.

Genes	Primer sequence (5′ to 3′)
TFAM	F: TTTCTCCGAAGCATGTGGGG
	R: CTTCAGCTTTTCCTGCGGTG
POLRMT	F: ACTCACCACAACAACCAAGACAAG
	R: CGTCCGTCAGCATGATGAACAC
TFB_2_M	F: AAGAATGCGGATGGAGAGTTACAAG
	R:GAACACCTGCTGACCAAGGAAC
MtDNA	F: GGTTCTTACTTCAGGGCCATCA
	R: TGATTAGACCCGTTACCATCGA

For analysis of mitochondrial DNA content, total DNA was extracted from tissues and cells using the Universal Genomic DNA kit (CW2298S, CWBIO, Beijing, China), and 10 ng of DNA was used for RT-qPCR analysis. MtDNA copy number was measured using the mitochondrial D-LOOP gene. Primer sequences (Shenggong, Shanghai, China) are shown in Table 1.

### Western blot

PC12 cells were washed 3 times with ice-cold PBS and lysed in RIPA buffer for 15 min on ice. Brain tissue (20 mg) was homogenized in RIPA buffer at 60 Hz, centrifuged at 15,000 rcf, and the supernatant collected as protein samples. Protein concentration was measured using a BCA kit (Basted). Equal protein amounts were separated by Sodium Dodecyl Sulfate Polyacrylamide Gel Electrophoresis (SDS-PAGE), transferred to Polyvinylidene fluoride (PVDF) membranes, and blocked with 5% skim milk at room temperature. Membranes were incubated with primary antibodies at 4 °C overnight, followed by HRP-conjugated secondary antibodies for 1 h at room temperature after TBST washing. Bands were visualized using a ChemiDoc XRS + system (Bio-Rad) and quantified with ImageJ (1.8.0). Tubulin served as the loading control.

### Statistical analysis

All results are expressed as mean ± SD. All data processing was performed using GraphPad Prism software, version 5.0. Results between treatments were compared using one-way Analysis of Variance (ANOVA) or two-way ANOVA. Results were considered significantly different at *P* < 0.05.

## Discussion

Given the brain’s high sensitivity to oxygen deprivation, hypoxia emerges as a pivotal factor in the pathogenesis of CIRI. During both the ischemic and reperfusion phases, there is a significant increase in ROS, which subsequently modulates vascular reactivity, damages vascular endothelial cells, and compromises the integrity of the blood-brain barrier [19]. Furthermore, ROS triggers lipid peroxidation of unsaturated fatty acids, precipitating the degradation and impairment of cellular and organellar membranes [20]. Consequently, these events culminate in a cascade of detrimental effects, including cerebral edema, inflammatory responses, neuronal apoptosis, and the expansion of the infarcted region, progressively exacerbating brain tissue damage and potentially leading to mortality [21].

ED, known as a classical neuroprotective agent and free radical scavenger, exerts its protective effects by neutralizing free radicals and reducing lipid peroxidation, thereby mitigating oxidative damage to cerebral, vascular endothelial, and neuronal cells [22, 23]. CAA, a natural phytochemical, exhibits multifaceted therapeutic properties, including anti-epileptic, anxiolytic, antidepressant, and antioxidative effects. However, the precise antioxidative mechanisms of CAA remain to be fully elucidated. In this study, we employed ED as a positive control to investigate the therapeutic mechanisms of CAA against oxidative stress induced by CIRI, with a particular focus on the potential interplay between CAA’s antioxidative properties and TFAM.

TFAM, a nuclear-encoded mitochondrial transcription factor, functions as a DNA-binding protein essential for the maintenance of mtDNA integrity [24]. It plays a dual role in stabilizing mtDNA and initiating its replication, making it indispensable for mtDNA metabolism. As a cornerstone of mitochondrial biogenesis, TFAM’s importance becomes particularly evident under oxidative stress, where ROS activates the PGC-1Α-NRF2 signaling pathway, leading to the upregulation of TFAM. This pathway acts as a critical antioxidant mechanism, enhancing cellular defenses against oxidative damage [25, 26]. In our experimental model, lentiviral-mediated knockdown of TFAM was achieved 24 h post ventricular injection, following established protocols [27, 28]. We observed a marked upregulation of TFAM expression in both brain tissue and PC12 cells following treatment with ED and CAA. Moreover, interference with TFAM exacerbated oxidative stress and tissue damage, as evidenced by elevated ROS levels and MDA content, further affirming the essential role of TFAM in mitigating oxidative stress.

Mitochondria, as the central hub of bioenergetic metabolism, are critically implicated in oxidative stress dynamics [13]. Our experimental assessments of mitochondrial function, including measurements of Ca^2+^ and ATP levels as well as mtDNA copy number, revealed that oxidative stress impairs mitochondrial function, as indicated by decreased ATP levels, increased Ca^2+^ concentrations, and a reduced mtDNA copy number in the model group. Treatment with ED and CAA ameliorated these mitochondrial dysfunctions, suggesting their potential in enhancing mitochondrial resilience against oxidative stress.

Further investigation into the TFAM signaling cascade revealed that TFAM is crucial for mtDNA transcription, thereby facilitating both mitochondrial genome replication and transcription. Specifically, TFAM initiates mtDNA transcription by binding to mitochondrial promoters, recruiting POLRMT, and subsequently interacting with TFB_2_M [13, 29]. This transcriptional activity enables the replacement of damaged mitochondria, thereby ameliorating oxidative stress-induced mitochondrial damage. Following treatment with ED and CAA, an upregulation in the expression of these transcription factors was noted, highlighting their protective pathways. Lentiviral-mediated knockdown of TFAM resulted in diminished expression of POLRMT and TFB_2_M across all groups, emphasizing TFAM’s critical role in mitochondrial transcription and biogenesis. Thus, we conclude that ED and CAA alleviate oxidative stress by modulating TFAM, thereby safeguarding mitochondrial integrity and mitigating oxidative damage through the regulatory interplay between TFAM and mitochondrial transcription biogenesis.

## Conclusions

TFAM plays a critical role in shielding the body from oxidative stress, including protecting mitochondrial function, alleviating oxidative stress damage, and promoting mitochondrial transcription biogenesis. The antioxidant mechanism of the body was further explored by the use of ED and CAA. These findings provide new research avenues for the prevention and treatment of ischemic stroke. We will continue to explore these studies in depth to elucidate the relevant antioxidant protective mechanisms.

## Supplementary information

supplemental material, WB blot

## Data Availability

The datasets support the findings of this study are available from the corresponding author upon reasonable request.
