# Supplementary figures and images for: TFAM signaling molecule alleviates mitochondrial damage of cerebral ischemia-reperfusion
Source: Cell Death Discov. 2026 Jan 8;12:83. doi: 10.1038/s41420-025-02930-x (PMC12877106; doi:10.1038/s41420-025-02930-x)

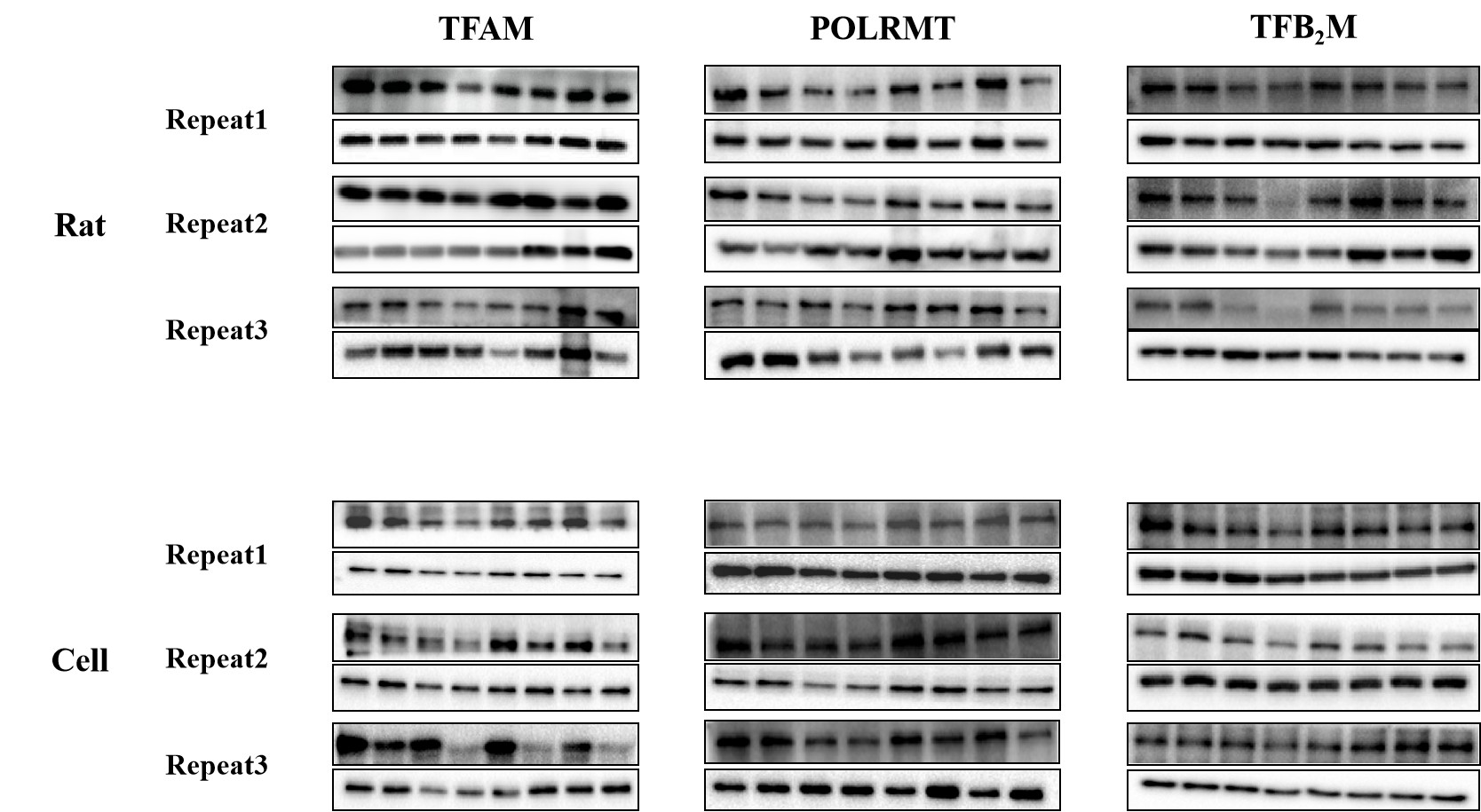

Supplement: Supplementary file 1 — supplemental material, WB blot [file 41420_2025_2930_MOESM1_ESM.jpg]
